# Bovine Blood Xenotransfusion as a Therapeutic Approach for the Treatment of Acute Blood Loss in Sheep

**DOI:** 10.3390/vetsci13040323

**Published:** 2026-03-27

**Authors:** José Felipe Napoleão Santos, Valesca Marques Melo, Samuel Barbosa Macedo, Rayara Silva de Freitas, Filipe Lima Costa, Aline Silva de Sant’ana, Ruan da Cruz Paulino, Antonio Humberto Hamad Minervino, Rejane Santos Sousa, Talyta Lins Nunes, Raimundo Alves Barrêto Junior

**Affiliations:** 1 Graduate Program in Animal Science, Universidade Federal Rural do Semi-Árido, Mossoró 59625-900, RN, Brazil; felipe_napoliao@hotmail.com (J.F.N.S.); ruanpaulino95@gmail.com (R.d.C.P.); 2 Undergraduate Program in Veterinary Medicine, Universidade Federal Rural do Semi-Árido, Mossoró 59625-900, RN, Brazil; valescaagm@gmail.com (V.M.M.); samuel.macedo@alunos.ufersa.edu.br (S.B.M.); rayarafreitasz@gmail.com (R.S.d.F.); filipelc09@gmail.com (F.L.C.); 3 Agricultural Sciences Coordination, Instituto Federal de Educação Ciência e Tecnologia do Espírito Santo, Montanha 29890-000, ES, Brazil; 4 Laboratory of Animal Health (LARSANA), Universidade Federal do Oeste do Pará, Santarém 68035-110, PA, Brazil; antonio.minervino@ufopa.edu.br; 5 Instituto de Estudos do Trópico Úmido (IETU), Universidade Federal do Sul e Sudeste do Pará, Xinguara 68555-000, PA, Brazil; rejane.sousa@unifesspa.edu.br; 6 Department of Animal Science, Universidade Federal Rural do Semi-Árido, Mossoró 59625-900, RN, Brazil; talyta.lins@ufersa.edu.br (T.L.N.); barreto@ufersa.edu.br (R.A.B.J.)

**Keywords:** anemia, critical care, hemotherapy, ruminants, transfusion medicine

## Abstract

Hemotherapy in small ruminants is indicated for several acute and chronic conditions; however, its clinical use is often limited by the difficulty in identifying suitable donors, particularly regarding blood volume availability and hematologic compatibility. Xenotransfusion in small ruminants with bovine blood may represent a practical alternative in emergency situations involving severe anemia when homologous donors are unavailable. This study evaluated the clinical, hematologic, biochemical, and blood gas responses of sheep subjected to acute blood loss followed by bovine whole blood xenotransfusion. Six healthy adult castrated male sheep (mean body weight 44.3 ± 7.2 kg) underwent removal of 40% of their estimated total blood volume. Parameters were assessed before hemorrhage induction (T0) and at times T30, T6h, T12h, T24h, T48h, T72h, T96h, T5d, T6d, T7d, T8d and T16d after transfusion. Acute blood loss significantly reduced packed cell volume and erythrocyte count at T0 (*p* < 0.05). After xenotransfusion, packed cell volume increased at T30min, T6h, and T12h and remained stable until T72h (*p* < 0.05), with progressive erythrocyte recovery and sustained macrocytosis. Total leukocyte count remained unchanged, whereas platelets increased at T7D (*p* < 0.05). Total protein decreased at T0 and subsequently increased. Transient elevations in urea, creatinine, glucose, pO_2_, and SO_2_ were observed (*p* < 0.05), without acid–base imbalance. Clinical parameters progressively stabilized, and no severe transfusion reactions occurred. Bovine whole blood xenotransfusion may represent a promising therapeutic alternative for sheep subjected to acute blood loss under the experimental conditions evaluated in this study. The procedure was associated with improvements in clinical, hematological, and biochemical parameters, and no severe transfusion reactions were observed during the monitoring period. These findings support the potential clinical applicability of this approach as an emergency intervention in situations where homologous donors are not readily available.

## Simple Summary

Severe blood loss can occur in sheep due to trauma, parasites, surgery, or other diseases, and it may become life-threatening when the number of red blood cells becomes too low. Blood transfusion is an important treatment in these situations because it helps restore blood volume and improve oxygen delivery to tissues. However, finding suitable blood donors among sheep is often difficult, especially in emergency conditions. This study investigated whether blood from cattle could be used as an alternative source for transfusion in sheep. Six healthy sheep were subjected to controlled blood loss to simulate severe anemia and then received whole blood from cattle. The animals were monitored through physical examinations and laboratory tests to evaluate their recovery and detect possible adverse reactions. The results showed that the transfusion improved important health indicators, including red blood cell levels and several clinical and biochemical parameters. The sheep gradually recovered after the procedure, and no severe transfusion reactions were observed. These findings suggest that using cattle blood may be a useful emergency treatment when sheep blood donors are not available. This approach could help veterinarians manage critical cases of blood loss in sheep and potentially improve survival in field conditions.

## 1. Introduction

Transfusion therapy is an established and increasingly utilized practice in veterinary medicine. Available modalities include whole blood, packed red blood cells, fresh or fresh-frozen plasma, platelet-rich plasma, and platelet concentrates [1]. Among these, whole blood transfusion remains the most commonly performed technique, with the primary objective of restoring oxygen-carrying capacity and tissue perfusion, as well as correcting severe anemia or coagulopathies, thereby stabilizing the patient until the underlying disease process can be identified and addressed.

In animal production practice, blood transfusion is typically considered an emergency intervention with transient and supportive effects. Its principal aim is the correction of hypovolemia, as mortality in these species is more frequently associated with circulatory collapse secondary to volume depletion than with impaired oxygen transport per se [2,3,4].

In small ruminants, transfusion therapy is primarily indicated in situations involving significant impairment of oxygen-carrying capacity. In clinical and hospital settings, this intervention is commonly employed in cases of severe anemia, acute hemorrhage, and various hematological disorders that result in a critical reduction in erythrocyte numbers and/or hemoglobin concentration [5]. Among the main conditions associated with the need for transfusion are severe anemia resulting from infestations with *Haemonchus contortus* [6]; acute hemorrhage caused by trauma, surgical procedures, or obstetric complications; hemoparasitic infections caused by agents such as *Anaplasma ovis*, *Babesia* spp., and *Mycoplasma ovis*, which induce erythrocyte destruction [7]; as well as anemias associated with nutritional deficiencies, particularly iron, copper, and vitamin B12 [8].

However, identifying a suitable blood donor in caprine and ovine production systems remains challenging. Limitations include insufficient donor body weight to provide adequate blood volume and variability in hematologic status that may preclude safe collection. These constraints underscore the need to investigate alternative transfusion strategies [9]. Given these limitations, investigating alternative transfusion strategies is essential to expand the available therapeutic options for these species.

In this context, blood transfusion between different species, known as xenotransfusion, has emerged as an area of increasing interest in veterinary medicine. This practice has been considered particularly relevant given the limitations in the availability of compatible donors, a situation commonly encountered in emergency settings. Initial studies and case reports have been more frequently described in dogs and cats [10]; however, the practice is not restricted to these species. Reports involving different animals have also been documented, including cattle and goats [11], as well as wildlife species such as foxes [12] and ferrets [13].

To date, there are no detailed experimental reports evaluating, under controlled conditions, the clinical and laboratory effects of bovine whole blood xenotransfusion in sheep, particularly in the context of acute blood loss. Therefore, the present study aimed to evaluate the clinical feasibility and safety of bovine whole blood xenotransfusion as a therapeutic intervention in sheep subjected to acute hemorrhage, as well as to characterize the associated clinical and laboratory responses in recipient animals. Furthermore, this study represents the first systematic experimental investigation reported in the scientific literature assessing this specific transfusion approach, contributing to the advancement of knowledge in ruminant internal medicine and to the development of novel therapeutic alternatives in veterinary medicine.

## 2. Materials and Methods

### 2.1. Study Location and Ethical Approval

The study was conducted at the Veterinary Internal Medicine Laboratory (LABMIV) of the Universidade Federal Rural do Semi-Árido (UFERSA). All experimental procedures were performed in accordance with the guidelines established by the Brazilian National Council for the Control of Animal Experimentation (CONCEA). The study protocol was reviewed and approved by the Institutional Animal Care and Use Committee (Comissão de Ética no Uso de Animais—CEUA) of UFERSA under approval number 17/2025.

### 2.2. Animal Selection and Management

Four clinically healthy, non-pregnant, non-lactating Holstein cows, approximately three years of age and weighing approximately 450 ± 50 kg (min–max: 400–450; *n* = 4), were selected as blood donors. The animals were sourced from the Dairy Cattle Sector of the Universidade Federal Rural do Semi-Árido (UFERSA), Mossoró, Brazil.

Six clinically healthy, castrated male sheep of mixed breed (no defined breed standard), approximately 1.5 years of age and with a mean initial body weight of 44.3 ± 7.2 kg (min–max: 38.7–55.9 kg; *n* = 6), were enrolled as transfusion recipients. None of the sheep had a prior history of blood transfusion.

The donor cattle were managed according to the standard husbandry practices of the university’s dairy unit. The recipient sheep were housed in collective pens (approximately 21 m^2^) within the animal confinement area of the Veterinary Internal Medicine Laboratory (LAMIV), equipped with shared feeders and automatic waterers. All animals were monitored and handled in accordance with animal welfare principles throughout the study. Animals were fed twice daily with Tifton hay and a commercial concentrate at a forage-to-concentrate ratio of 70:30 on an as-fed basis. Mineral supplementation formulated for sheep Masterfós^®^ (Master Alimentos Ltda., Rio Pomba, MG, Brazil)and water were provided ad libitum.

Prior to the experimental period, all recipient animals underwent comprehensive clinical evaluation, including physical examination, complete blood count, serum biochemical profiling, fecal parasitological examination, and urinalysis to confirm health status. Subsequently, animals were dewormed and treated for endoparasites with ivermectin (Provermin^®^ (Indubras^®^, Rio Largo, AL, Brazil)., administered according to the manufacturer’s recommended dosage) and vaccinated against clostridial diseases (Excell 10, Dechra^®^, Londrina, Brazil). A booster vaccination was administered 30 days later during the adaptation period.

### 2.3. Experimental Design

The experimental period lasted 90 days, consisting of 45 days for animal adaptation to the facilities and 45 days dedicated to data collection. The adaptation period began in November 2025.

### 2.4. Experimental Protocol

The six recipient sheep were catheterized and subjected to acute blood loss via the external jugular vein. Twenty-four hours later, they received a transfusion of bovine whole blood immediately after donor blood collection. Clinical, hematologic, biochemical, urinary, and blood gas parameters were subsequently evaluated at predetermined time points.

Prior to transfusion, crossmatching tests were performed according to [14], and only donor–recipient pairs without evidence of incompatibility were selected for xenotransfusion.

#### 2.4.1. Blood Compatibility Testing

One week prior to xenotransfusion, blood compatibility testing was performed between three bovine donors and six ovine recipients using both major and minor crossmatch techniques. Blood samples were collected by jugular venipuncture into anticoagulant-containing tubes, properly identified, and processed in the laboratory.

Following centrifugation, plasma was separated and transferred to sterile tubes. The packed red blood cells (RBCs) were washed three consecutive times with isotonic saline solution (0.9% NaCl) by sequential resuspension, centrifugation, and supernatant removal to eliminate residual plasma proteins. A final RBC suspension was prepared for use in crossmatching reactions.

For the major crossmatch, 50 µL of donor washed RBCs were combined with 100 µL of recipient serum. For the minor crossmatch, 100 µL of donor serum were combined with 50 µL of recipient washed RBCs. Given that each bovine donor was tested against all six ovine recipients, 18 major and 18 minor crossmatches were performed, totaling 36 reactions.

Mixtures were gently homogenized, maintained at room temperature for 15 min, and subsequently incubated in a water bath at 37 °C for an additional 15 min. Samples were then centrifuged, and the supernatant was macroscopically evaluated for hemolysis. Agglutination was assessed on a tilted slide and confirmed by light microscopy. No incompatibility reactions were observed in the tests performed.

#### 2.4.2. Induction of Acute Blood Loss

Recipient sheep underwent trichotomy and aseptic preparation of both right and left external jugular veins. Phlebotomy was then performed, removing 40% of the total blood volume, calculated based on an estimated blood volume corresponding to 8% of body weight. The removal of 40% of the estimated blood volume was selected to reproduce a condition of severe acute anemia, which may occur in several emergency clinical scenarios in sheep. This level of blood loss was induced in a controlled manner and under continuous monitoring in order to simulate a severe clinical condition with minimal impact on the animals. Blood loss of this magnitude is known to induce multiple systemic alterations [9].

During the procedure, animals were physically restrained in a standing position on a handling table. After blood removal, sheep were returned to their collective pens and monitored for 24 h prior to xenotransfusion.

#### 2.4.3. Bovine Blood Collection

Donor cows underwent aseptic preparation of the external jugular vein for blood collection. Whole blood was collected into a CPDA-1 anticoagulant with a capacity of up to 450 mL. The transfusion volume was calculated according to recipient body weight, establishing a dose of 20 mL of whole blood per kilogram of recipient body weight, while respecting safe collection limits for bovine donors. The volume collected from the bovine donors corresponded to the calculated amount required to compensate for the blood loss in the recipient sheep.

#### 2.4.4. Xenotransfusion Procedure

Twenty-four hours after induction of acute blood loss, sheep received bovine whole blood xenotransfusion at a dose of 20 mL/kg. During the first 30 min, blood was administered at a rate of 0.25 mL/kg over 30 min. In the absence of transfusion reactions, the infusion rate was increased to 20 mL/kg/h.

Emergency medication remained readily available throughout the procedure.

### 2.5. Experimental Time Points

Animals underwent physical examination and sample collection at the following time points: TB (24 h prior to acute blood loss); T0 (24 h after induction of anemia and immediately before transfusion); T30 (30 min after transfusion); T6 (6 h after transfusion); T12 (12 h after transfusion); T24 (24 h after transfusion); T48 (48 h after transfusion); T72 (72 h after transfusion); T96 (96 h after transfusion); T5d (5 days after transfusion); T6d (6 days after transfusion); T7 (7 days after transfusion); T8d (8 days after transfusion); T16d (16 days after transfusion).

### 2.6. Evaluated Parameters

#### 2.6.1. Physical Examination

At each predetermined time point, a complete physical examination was performed, including heart rate, respiratory rate, ruminal motility, and rectal temperature.

Heart rate was determined by auscultation for one minute with the animal at rest. Respiratory rate was assessed by counting thoracic movements for one minute. Ruminal contractions were evaluated over a two-minute period using a stethoscope. Rectal temperature was measured using a digital clinical thermometer [15].

#### 2.6.2. Hematologic Evaluation

Venous blood samples were collected into EDTA-containing tubes for hematologic analysis. The following parameters were determined: packed cell volume (PCV), erythrocyte count, total hemoglobin concentration, mean corpuscular volume (MCV), mean corpuscular hemoglobin concentration (MCHC), total leukocyte count, and platelet count. Analyses were performed using a semi-automated hematology analyzer (Urit BH-70P, MBLab^®^ Guilin, China).

#### 2.6.3. Biochemical Evaluation

For biochemical analysis, blood samples were collected into vacuum tubes without anticoagulant, centrifuged at 1500 rpm for 10 min, and serum was immediately analyzed.

Serum concentrations of total protein (TP), albumin, aspartate aminotransferase (AST), lactate dehydrogenase (LDH), gamma-glutamyl transferase (GGT), creatine kinase (CK), urea, and creatinine were measured using commercial reagents (VIDA, Biotecnologia^®^, Minas gerais, Brazil) and analyzed with a Human Star 80 biochemical analyzer (Human GmbH, Human Diagnostcs^®^, Wiesbaden, Germany).

Total and indirect bilirubin concentrations were determined by a colorimetric method using commercial reagents (VIDA^®^, Brazil) in a semi-automated biochemical analyzer (Sinnoawa SX-3000, Sinnowa Medical Science & Technology Co., Ltd.^®^, Nanjing, China).

For glucose determination, blood samples were collected into fluoride-containing tubes, centrifuged to obtain plasma, and analyzed using commercial kits in the same semi-automated analyzer (Sinnowa SX-3000M).

#### 2.6.4. Blood Gas Analysis

Venous blood samples were collected into lithium heparin syringes for blood gas analysis. Measurements were performed using a portable blood gas analyzer (Seamaty, Seamaty Médica^®^, Chengdu, China) at TB, T0, T6, and T12.

The following parameters were measured: pH, partial pressures of oxygen (pO_2_) and carbon dioxide (pCO_2_), oxygen saturation (SO_2_), bicarbonate concentration (HCO_3_^−^), and base excess (BE).

### 2.7. Statistical Analysis

Data were analyzed using linear mixed-effects models, with *time* as a fixed effect and *animal* as a random effect, thereby accounting for the correlation structure inherent to repeated measures obtained from the same individual over time. Variance components were estimated using restricted maximum likelihood (REML). Degrees of freedom were adjusted using the Kenward–Roger method, appropriate for small sample sizes and repeated-measures designs. The overall effect of time on response variables was initially assessed using the F-test from the mixed model. When a significant effect was detected (*p* < 0.05), multiple comparisons among time points were performed using Tukey’s procedure with pairwise adjustment to control for type I error. Interindividual variability was incorporated by including animal as a random effect in all models. All statistical analyses were conducted using Minitab^®^ software, version 18, with a significance level set at 5% (α = 0.05).

## 3. Results

During the experimental period, no clinical transfusion reactions were observed in the recipient sheep following bovine whole blood xenotransfusion. The animals were continuously monitored during and after the procedure, and no alterations compatible with acute transfusion reactions were detected, such as hyperthermia, marked tachycardia, dyspnea, muscle tremors, hemoglobinuria, or signs of shock.

The method used to induce acute blood loss in sheep proved effective, allowing the removal of 40% of the estimated total blood volume. The mean volume withdrawn was 1.413 ± 0.24 L (mean ± SD). Subsequently, the animals received a mean volume of 0.885 ± 0.15 L of bovine whole blood from donors considered compatible based on prior crossmatching tests. No severe transfusion reactions were observed in any of the animals throughout the study period.

Regarding clinical variables, a significant decrease in heart rate was observed at T72h and T16D compared with T0 (*p* < 0.05). Respiratory rate was significantly increased at T48h, T96h, and T5D compared with T16D (*p* < 0.05).

Rectal temperature was significantly lower at TB compared with T96h and T5D (*p* < 0.05). No significant differences were observed in ruminal motility across experimental time points (*p* > 0.05) (Table 1).

Regarding hematologic variables, a significant decrease in packed cell volume (PCV) was observed at T0 compared with TB (*p* < 0.05), confirming the establishment of acute anemia. Following xenotransfusion, PCV values significantly increased at T30, T6, and T12 compared with T0 (*p* < 0.05). Between T24 and T48, PCV remained stable and was maintained through T72.

Red blood cell (RBC) count similarly declined at T0 compared with TB. From T30 onward, a significant increase was observed, persisting through T72 (*p* < 0.05) (Table 2).

Mean corpuscular volume (MCV) showed a significant increase from T30 through T16D compared with TB (*p* < 0.05), indicating maintenance of a macrocytic profile. Total leukocyte count did not differ significantly across time points (*p* > 0.05). Platelet count showed a significant decrease from T0 through T96 compared with TB (*p* < 0.05) (Table 2).

With respect to biochemical variables, total protein concentration significantly decreased at T0 compared with TB (*p* < 0.05). Following xenotransfusion, total protein levels increased significantly from T30 through T16D (*p* < 0.05). Serum albumin concentrations differed significantly at T5D compared with T0, T30, T6, T12, T24, T48, T7D, T8D, and T16D (*p* < 0.05). Serum urea concentrations were significantly increased at T30, T24, and T5D compared with TB (*p* < 0.05). Creatinine levels were elevated at T0, T30, and T5D relative to TB (*p* < 0.05). Blood glucose concentrations were significantly higher at T0, T30, and T5D compared with TB (*p* < 0.05) (Table 3).

Regarding blood gas parameters (Table 4), a significant reduction in partial pressure of oxygen (pO_2_) was observed at T6 compared with TB (*p* < 0.05). Oxygen saturation (SO_2_) was also significantly decreased at T6 relative to all other evaluated time points (*p* < 0.05). No significant differences were observed over time for pH, bicarbonate concentration (HCO_3_^−^), or partial pressure of carbon dioxide (pCO_2_) (*p* > 0.05).

## 4. Discussion

This study is the first to experimentally and systematically investigate bovine whole blood xenotransfusion as a therapeutic alternative for the correction of acute blood loss in sheep. The unavailability of an ideal donor for clinically compromised animals is a frequent challenge in small ruminant practice, particularly under field conditions, whether due to limitations in donor blood volume, inadequate hematological status for safe collection, or the absence of established blood banks for small ruminants. In this context, the use of bovine blood as a potential xenogeneic source represents an innovative strategy with immediate practical applicability, especially in situations in which homologous transfusion is not feasible.

In addition to the longitudinal assessment of clinical, hematological, biochemical, and blood gas responses, this study contributes to understanding the physiological tolerability and risks associated with interspecies transfusion in ruminants. The findings may support the development of emergency protocols, guide future investigations, and expand current knowledge on alternative transfusion therapies, representing a novel and relevant contribution to experimental and clinical veterinary medicine.

The absence of severe transfusion reactions in sheep subjected to bovine xenotransfusion is a clinically relevant finding, given the inherent immunological potential of interspecies transfusions. Although incompatibility between erythrocyte antigens could theoretically trigger acute hemolytic reactions and significant systemic manifestations, the lack of severe clinical signs suggests that, under the experimental conditions adopted, the antigenic load and transfusion management were insufficient to induce an immediate exaggerated immune response. This outcome may be associated with low titers of naturally occurring antibodies, gradual clearance of heterologous erythrocytes by the mononuclear phagocyte system, and adherence to appropriate blood collection and administration protocols. Nevertheless, the possibility of mild reactions or progressive extravascular hemolysis cannot be excluded, and these findings should be interpreted cautiously regarding clinical applicability and the need for rigorous post-transfusion monitoring.

The initial increase in heart rate observed at T0 is physiologically consistent with the expected compensatory response to acute anemia and hypovolemia, in which sympathetic nervous system activation, catecholamine release, and increased cardiac output act to preserve tissue perfusion and oxygen transport [16,17]. The progressive reduction in heart rate following xenotransfusion, particularly at T72h and T16D, suggests improvement in hemodynamic status and tissue oxygenation in recipient animals, possibly resulting from partial restoration of circulating volume and oxygen-carrying capacity provided by transfused bovine erythrocytes. Similar patterns have been described in ruminants undergoing blood transfusion, in which cardiovascular stabilization occurs after volume replacement, even when transfused erythrocyte survival is limited [16,18,19].

The increase in respiratory rate observed at intermediate time points (T48h, T96h, and T5D) may be associated with multiple pathophysiological mechanisms. Initially, tachypnea may reflect a compensatory response to residual anemia, as reduced oxygen-carrying capacity stimulates peripheral chemoreceptors, increasing pulmonary ventilation [20]. Additionally, inflammatory responses secondary to heterologous transfusion may have contributed to transient respiratory changes, since xenotransfusion can induce moderate immune activation and release of inflammatory mediators, even in the absence of severe clinical manifestations [21,22]. The reduction in respiratory rate at the final time point (T16D) suggests restoration of physiological homeostasis, possibly associated with progressive hematological recovery [16].

Regarding rectal temperature, the increase observed at T96h and T5D compared with baseline may be attributed to post-transfusion inflammatory responses. Febrile non-hemolytic reactions are among the most common complications of transfusion therapy and are associated with the release of inflammatory cytokines and interactions between donor and recipient leukocytes [21,22]. In the context of xenotransfusion, this response may be more pronounced due to greater antigenic disparity between species, although it generally remains self-limiting and without severe clinical consequences.

Following xenotransfusion, a significant increase in packed cell volume was observed at T30min, T6h, and T12h compared with T0, followed by stabilization between T24h and T72h. This pattern suggests that bovine whole blood transfusion effectively promoted plasma volume expansion and a transient increase in circulating erythrocyte mass in recipient sheep. In ruminants, the primary therapeutic goal of blood transfusion is restoration of circulating volume and tissue perfusion, whereas transfused erythrocyte survival is often limited, particularly in the presence of antigenic incompatibility, as expected in xenotransfusion [23]. Therefore, hematocrit stabilization up to T72h may reflect both temporary persistence of bovine erythrocytes in circulation and fluid redistribution following volume replacement.

Red blood cell count showed a pattern similar to packed cell volume, with a significant decrease at T0 and progressive recovery, evident by T48h. This finding reinforces the initial hematological efficacy of xenotransfusion and is consistent with studies demonstrating that blood transfusion in ruminants results in rapid clinical improvement, even when erythrocyte persistence is transient [11].

The significant increase in mean corpuscular volume (MCV) from T30min to T16D relative to baseline indicates maintenance of a macrocytic profile throughout the experimental period. Two main mechanisms may explain this finding. First, transfused bovine erythrocytes are larger than ovine erythrocytes, directly contributing to increased circulating MCV. Second, regenerative response to anemia, characterized by reticulocyte release, may have contributed, as reticulocytes exhibit greater cell volume than mature erythrocytes [24]. Thus, persistence of a macrocytic profile suggests a combined contribution of heterologous transfusion and regenerative erythropoiesis in recipient animals.

The absence of significant differences in total leukocyte count among evaluated time points indicates that xenotransfusion did not induce a clinically relevant systemic inflammatory response or marked leukocytosis. Although heterologous transfusions may potentially trigger immunological reactions, severe clinical responses are uncommon when moderate volumes are administered and when there is no prior recipient sensitization [21].

Regarding platelets, a significant increase was observed at T7D compared with baseline, characterizing a delayed hematological response following acute blood loss and subsequent xenotransfusion. This finding is consistent with reactive thrombocytosis secondary to hemorrhage, resulting from bone marrow activation stimulated by tissue hypoxia and inflammatory mediators, with increased megakaryocytic production [24]. In ruminants, this compensatory response typically occurs between the fifth and seventh day after hemorrhagic insult, coinciding with the period observed in this study.

The biochemical alterations observed reflect the pathophysiological responses triggered by induced acute anemia, the transfusion procedure, and subsequent compensatory metabolic mechanisms. The significant reduction in total protein at T0 compared with baseline (TB) can be primarily explained by acute blood loss, which reduces both cellular and plasma fractions of circulating blood, as well as by redistribution of fluids from the interstitial to the intravascular compartment—a typical compensatory mechanism in hemorrhagic conditions responsible for hemodilution and decreased serum protein concentrations [16,25].

After xenotransfusion, the progressive increase in total protein between T30 and T16D suggests recovery of plasma volume, possible contribution of plasma proteins from transfused bovine blood, and participation of a moderate systemic inflammatory response induced by the procedure, considering that acute-phase proteins may increase total serum concentrations [18]. Additionally, improvement in hemodynamic status after volume replacement may have favored the resumption of hepatic protein synthesis, contributing to gradual normalization of observed values.

Regarding albumin, the significant difference observed at T5D compared with other time points may reflect transient alterations in the balance among hepatic synthesis, vascular redistribution, and inflammatory status. Albumin is a negative acute-phase protein and may vary in response to physiological stress, inflammation, and hemodynamic changes [26]. The increase observed at T5D may be associated with metabolic recovery following the initial adaptation period to anemia and transfusion, as well as normalization of circulatory status.

Serum urea concentrations were significantly increased at T24h, T48h, T72h, and T5D compared with baseline. This finding may be explained by increased protein catabolism secondary to metabolic stress induced by acute anemia, as well as transient reduction in renal perfusion during the initial hypovolemic period [22]. In ruminants, moderate increases in urea may occur in prerenal hypoperfusion states and are often reversible following hemodynamic stabilization [9,27].

Creatinine increased at T30min compared with baseline, likely reflecting a transient decrease in glomerular filtration rate resulting from initial hypovolemia and circulatory redistribution during hemorrhagic shock. In acute anemia, renal perfusion may be temporarily compromised, leading to mild creatinine elevations that tend to normalize after restoration of circulating volume [27]. The absence of persistent increases suggests that no significant structural renal injury occurred in the evaluated animals.

Higher glucose concentrations observed at T30min compared with TB can be attributed to the neuroendocrine response to physiological stress. Acute anemia and experimental procedures activate the hypothalamic–pituitary–adrenal axis, promoting cortisol and catecholamine release, which stimulate hepatic glycogenolysis and gluconeogenesis, resulting in transient hyperglycemia [20,28,29]. Additionally, administration of transfused blood may contribute to temporary metabolic changes due to the supply of energetic substrates and inflammatory mediators. Subsequent glycemic stabilization suggests recovery of metabolic balance.

The blood gas parameters evaluated in this study demonstrated specific alterations consistent with the pathophysiology of acute anemia and with respiratory and hemodynamic adaptations following transfusion [20,29]. The significant reduction in partial pressure of oxygen (pO_2_) at T6h compared with baseline (TB), together with decreased oxygen saturation (SO_2_) at the same time point, suggests transient impairment of blood oxygenation after the transfusion procedure. Conversely, the absence of significant differences over time in pH, bicarbonate (HCO_3_^−^), and partial pressure of carbon dioxide (pCO_2_) indicates maintenance of systemic acid–base balance throughout the experimental period [25].

The reduction in pO_2_ and SO_2_ at T6h may be explained by physiological mechanisms related to acute anemia and early circulatory adaptation following transfusion. Reduced erythrocyte mass decreases oxygen-carrying capacity, potentially resulting in lower arterial oxygen availability even when pulmonary ventilation remains adequate [16,22]. Furthermore, in the context of xenotransfusion, the presence of bovine erythrocytes in ovine circulation may temporarily influence oxygen delivery dynamics, as interspecies differences in hemoglobin oxygen affinity may alter the oxyhemoglobin dissociation curve [30].

Although alterations were observed during the immediate post-transfusion period, these changes were transient and consistent with the physiological adaptations expected following acute anemia and volume replacement, without resulting in adverse clinical consequences. Throughout the experimental period, a progressive stabilization of both clinical status and laboratory parameters was observed, indicating restoration of systemic homeostasis. Thus, the findings indicate that this procedure may represent a promising emergency strategy, particularly in situations where immediate availability of same-species donors is not feasible.

## 5. Conclusions

Bovine whole blood xenotransfusion may represent a promising therapeutic alternative for sheep subjected to acute blood loss under the experimental conditions evaluated in this study. The procedure was associated with improvements in clinical, hematological, and biochemical parameters, and no severe transfusion reactions were observed during the monitoring period. These findings support the potential clinical applicability of this approach as an emergency intervention in situations where homologous donors are not readily available.

## 6. Perspectives

As this is a pioneering study, further investigations are required to evaluate the feasibility of this approach and its clinical applicability under diverse experimental and field conditions, in order to consolidate its safety and efficacy. Future studies should also investigate the duration of bovine erythrocyte survival in ovine circulation, the potential immunological responses associated with interspecies transfusion, and the clinical outcomes of repeated or larger-volume xenotransfusions. Additionally, evaluating this strategy in different clinical scenarios of naturally occurring anemia may help to better define its practical applicability as an emergency therapeutic option in small ruminant medicine.

## Figures and Tables

**Table 1:** Mean values and standard deviation of heart rate (HR), respiratory rate (RR), rectal temperature (T), and ruminal movements (RM) in sheep undergoing bovine whole blood xenotransfusion.

	Variables			
Moments				
	HR (90–115 bpm)	RR (20–30 mpm)	T (°C) (38.5–40.0)	RM (mov/2 min)
TB	112 ± 19 ^AB^	51 ± 22 ^AB^	38.8 ± 0.43 ^C^	1.2 ± 0.7
T0	134 ± 16 ^A^	69 ± 36 ^AB^	39.0 ± 0.23 ^BC^	1.5 ± 0.5
T30	105 ± 19 ^AB^	71 ± 22 ^AB^	39.2 ± 0.09 ^BC^	1.2 ± 0.7
T6	102 ± 18 ^AB^	48 ± 19 ^AB^	39.4 ± 0.11 ^BC^	1.0 ± 0.6
T12	105 ± 18 ^AB^	46 ± 29 ^AB^	39.3 ± 0.25 ^BC^	1.1 ± 0.6
T24	108 ± 16 ^AB^	66 ± 23 ^AB^	39.4 ± 0.20 ^BC^	1.0 ± 0.8
T48	104 ± 12 ^AB^	83 ± 24 ^A^	39.3 ± 0.20 ^BC^	1.1 ± 0.6
T72	93 ± 11 ^B^	73 ± 32 ^AB^	39.0 ± 0.24 ^BC^	0.8 ± 0.7
T96	102 ± 28 ^AB^	81 ± 37 ^A^	40.2 ± 0.79 ^A^	1.2 ± 0.7
T5D	106 ± 17 ^AB^	81 ± 41 ^A^	39.6 ± 0.45 ^B^	1.5 ± 0.8
T6D	113 ± 18 ^AB^	67 ± 25 ^AB^	39.5 ± 0.37 ^BC^	0.8 ± 0.6
T7D	107 ± 13 ^AB^	49 ± 27 ^AB^	39.0 ± 0.53 ^BC^	0.8 ± 0.7
T8D	117 ± 13 ^AB^	57 ± 27 ^AB^	39.1 ± 0.38 ^BC^	1.0 ± 0.8
T16D	95 ± 16 ^B^	42 ± 24 ^B^	39.0 ± 0.38 ^BC^	1.8 ± 1.7

TB (24 h before acute blood loss); T0 (24 h after induction of anemia and immediately prior to transfusion); T30 (30 min after transfusion); T6 (6 h after transfusion); T12 (12 h after transfusion); T24 (24 h after transfusion); T48 (48 h after transfusion); T72 (72 h after transfusion); T96 (96 h after transfusion); T5 (5 days after transfusion); T6D (6 days after transfusion); T7 (7 days after transfusion); T8 (8 days after transfusion); and T16 (16 days after transfusion). Values are expressed as mean ± standard deviation (SD). Different uppercase letters within the same column indicate statistically significant differences between moments (*p* < 0.05).

**Table 2:** Mean values and standard deviation of packed cell volume (PCV), red blood cell count (RBC), mean corpuscular volume (MCV), total leukocyte count (WBC), and platelet count in sheep undergoing bovine whole blood xenotransfusion.

	Variables				
Moments					
	Packed Cell Volume (L/L)	Red Blood Cells (×10^12^/L)	Mean Corpuscular Volume (fL)	Leukocytes (×10^6^/L)	Platelets (×10^9^/L)
TB	33.5 ± 4.63 ^A^	9.7 ± 0.5 ^A^	34.30 ± 2.6 ^BC^	10.83 ± 2.4 ^AB^	409.50 ± 82.8 ^BCD^
T0	15.3 ± 3.09 ^F^	4.9 ± 0.9 ^D^	31.72 ± 2.8 ^C^	9.63 ± 1.1 ^AB^	207.67 ± 64.9 ^CD^
T30	19.7 ± 1.70 ^DEF^	5.3 ± 0.7 ^CD^	38.72 ± 1.3 ^A^	8.83 ± 1.8 ^AB^	142.67 ± 66.6 ^D^
T6	23.5 ± 2.06 ^BCDE^	6.0 ± 0.4 ^BCD^	38.82 ± 1.6 ^A^	8.27 ± 0.5 ^AB^	207.00 ± 117.6 ^CD^
T12	23.6 ± 1.80 ^BCDE^	6.1 ± 0.6 ^BCD^	38.77 ± 1.7 ^A^	8.48 ± 1.2 ^AB^	208.17 ± 89.9 ^CD^
T24	23.7 ± 2.97 ^BCD^	6.1 ± 0.8 ^BCD^	38.73 ± 1.5 ^A^	8.12 ± 1.9 ^AB^	210.33 ± 84.6 ^BCD^
T48	25.2 ± 2.29 ^BC^	6.4 ± 0.3 ^BC^	38.40 ± 2.3 ^A^	7.70 ± 2.1 ^B^	272.33 ± 97.7 ^BCD^
T72	22.9 ± 1.04 ^CDE^	5.5 ± 0.9 ^CD^	38.77 ± 1.3 ^A^	7.88 ± 2.0 ^B^	276.25 ± 138.1 ^BCD^
T96	19.7 ± 2.39 ^DEF^	5.1 ± 0.3 ^CD^	37.47 ± 2.7 ^A^	12.92 ± 2.8 ^AB^	372.07 ± 34.7 ^BCD^
T5D	19.4 ± 4.84 ^DEF^	5.2 ± 0.8 ^CD^	36.85 ± 4.0 ^AB^	13.60 ± 3.2 ^A^	417.50 ± 238.2 ^ABCD^
T6D	19.1 ± 5.01 ^DEF^	5.0 ± 0.9 ^D^	37.93 ± 3.3 ^A^	12.15 ± 5.2 ^AB^	473.00 ± 287.6 ^ABC^
T7D	18.7 ± 3.59 ^EF^	5.4 ± 1.2 ^CD^	38.28 ± 3.6 ^A^	12.57 ± 5.8 ^AB^	606.67 ± 266.8 ^A^
T8D	20.6 ± 2.03 ^CDE^	5.4 ± 0.3 ^CD^	38.95 ± 3.4 ^A^	10.73 ± 0.9 ^AB^	518.83 ± 161.8 ^AB^
T16D	28.3 ± 4.32 ^B^	7.1 ± 1.0 ^B^	39.93 ± 2.1 ^A^	9.67 ± 4.8 ^AB^	515.83 ± 270.0 ^ABC^

TB (24 h before acute blood loss); T0 (24 h after induction of anemia and immediately prior to transfusion); T30 (30 min after transfusion); T6 (6 h after transfusion); T12 (12 h after transfusion); T24 (24 h after transfusion); T48 (48 h after transfusion); T72 (72 h after transfusion); T96 (96 h after transfusion); T5 (5 days after transfusion); T6D (6 days after transfusion); T7 (7 days after transfusion); T8 (8 days after transfusion); and T16 (16 days after transfusion). Values are expressed as mean ± standard deviation (SD). Different uppercase letters within the same column indicate statistically significant differences between moments (*p* < 0.05).

**Table 3:** Mean values and standard deviation of blood biochemical variables of sheep submitted to xenotransfusion.

	Variables				
Moments					
	Total Protein (g/dL)	Albumin (g/dL)	Urea (mg/dL)	Creatinine (mg/dL)	GLU (mmol/L)
TB	6.9 ± 1.0 ^AB^	3.4 ± 0.1 ^ABC^	21.5 ± 5.9 ^D^	0.9 ± 0.2 ^B^	68.7 ± 6.2 ^B^
T0	5.4 ± 0.3 ^B^	2.7 ± 0.6 ^BC^	38.1 ± 8.8 ^ABCD^	1.0 ± 0.1 ^AB^	74.0 ± 7.1 ^AB^
T30	6.2 ± 1.0 ^AB^	3.1 ± 0.3 ^BC^	32.8 ± 10.6 ^ABCD^	1.0 ± 0.1 ^A^	91.8 ± 18.0 ^A^
T6	7.3 ± 0.8 ^A^	2.7 ± 1.25 ^BC^	35.3 ± 10.9 ^ABCD^	0.9 ± 0.2 ^B^	67.2 ± 18.6 ^B^
T12	7.6 ± 0.9 ^A^	2.8 ± 1.0 ^BC^	33.1 ± 9.4 ^ABCD^	1.0 ± 0.09 ^B^	68.2 ± 16.6 ^B^
T24	7.1 ± 0.4 ^A^	2.2 ± 0.7 ^BC^	50.6 ± 13.8 ^A^	1.0 ± 0.1 ^B^	67.8 ± 9.4 ^B^
T48	7.4 ± 1.8 ^A^	2.7 ± 0.4 ^BC^	50.6 ± 10.8 ^A^	1.0 ± 0.2 ^B^	67.2 ± 4.8 ^B^
T72	7.5 ± 0.4 ^A^	3.6 ± 0.3 ^AB^	42.3 ± 11.7 ^ABC^	1.1 ± 0.2 ^B^	70.4 ± 7.4 ^B^
T96	7.7 ± 0.4 ^A^	3.6 ± 0.5 ^AB^	39.8 ± 5.2 ^ABCD^	1.1 ± 0.1 ^B^	70.1 ± 7.7 ^B^
T5D	7.5 ± 0.5 ^A^	4.1 ± 0.4 ^A^	44.0 ± 10.7 ^AB^	1.0 ± 0.1 ^AB^	78.0 ± 12.4 ^AB^
T6D	6.4 ± 1.3 ^AB^	3.4 ± 0.3 ^ABC^	37.8 ± 7.2 ^ABCD^	0.9 ± 0.08 ^B^	66.8 ± 5.1 ^B^
T7D	6.8 ± 0.6 ^AB^	2.7 ± 0.2 ^BC^	32.1 ± 4.0 ^BCD^	0.9 ± 0.1 ^B^	62.5 ± 7.2 ^B^
T8D	7.1 ± 0.5 ^A^	2.5 ± 0.2 ^C^	27.6 ± 6.31 ^BCD^	0.8 ± 0.1 ^B^	59.2 ± 5.5 ^B^
T16D	7.8 ± 0.2 ^A^	2.7 ± 0.2 ^BC^	25.0 ± 6.0 ^CD^	1.0 ± 0.08 ^B^	58.9 ± 13.3 ^B^

TB (24 h before acute blood loss); T0 (24 h after induction of anemia and immediately prior to transfusion); T30 (30 min after transfusion); T6 (6 h after transfusion); T12 (12 h after transfusion); T24 (24 h after transfusion); T48 (48 h after transfusion); T72 (72 h after transfusion); T96 (96 h after transfusion); T5 (5 days after transfusion); T6D (6 days after transfusion); T7 (7 days after transfusion); T8 (8 days after transfusion); and T16 (16 days after transfusion). Values are expressed as mean ± standard deviation (SD). Different uppercase letters within the same column indicate statistically significant differences between moments (*p* < 0.05).

**Table 4:** Mean values and standard deviation of blood gases from sheep undergoing xenotransfusion.

	Variables				
Moments					
	pH	HCO _3_ (mmol/L)	pCO _2_ (mmHg)	pO_2_ (mmHg)	SO_2_ (%)
TB	7.45 ± 0.56	25.32 ± 4.17	36.66 ± 1.83	36.0 ± 8.03 ^A^	70.32 ± 8.72 ^A^
T0	7.46 ± 0.47	26.08 ± 3.33	36.24 ± 3.16	34.4 ± 4.93 ^AB^	69.16 ± 10.61 ^A^
T6	7.46 ± 0.45	28.06 ± 2.62	38.42 ± 2.90	28.8 ± 4.38 ^B^	58.50 ± 8.89 ^B^
T12	7.42 ± 0.83	25.30 ± 4.79	37.46 ± 2.42	34.2 ± 6.53 ^AB^	66.64 ± 9.27 ^AB^

TB (24 h before acute blood loss); T0 (24 h after induction of anemia and immediately prior to transfusion); T6 (6 h after transfusion); T12 (12 h after transfusion). Values are expressed as mean ± standard deviation (SD). Different uppercase letters within the same column indicate statistically significant differences between moments (*p* < 0.05).

## Data Availability

The original contributions presented in this study are included in the article. Further inquiries can be directed to the corresponding author.
